# Infantile Hepatic Hemangioma: A Novel Approach Using Propranolol and Transarterial Embolization

**DOI:** 10.7759/cureus.66507

**Published:** 2024-08-09

**Authors:** Divyanshi Kaplish, Jayant D Vagha, Sham Lohiya, Shailesh Wandile, Sri Sita Naga Sai Priya K.

**Affiliations:** 1 Pediatrics, Jawaharlal Nehru Medical College, Datta Meghe Institute of Higher Education and Research, Wardha, IND

**Keywords:** consumptive hypothyroidism, hepatic hemangioma, propranolol, transarterial embolisation, hepatic hemangioendothelioma

## Abstract

Benign vascular tumors, or hemangiomas, are common in young children. The most frequent way to identify them on the skin is as bright red surface lesions, although they can also be detected deeper as subcutaneous lesions. Visceral involvement, particularly of the liver, is commonly observed in patients with multiple cutaneous hemangiomas. Since most hemangiomas are self-limited, they can be clinically monitored. Despite this, hepatic hemangiomas can result in significant consequences, such as severe hepatomegaly, which can induce abdominal compartment syndrome, inadequate ventilation, and renal vein compression, as well as significant arteriovenous shunts that compromise the functioning of the heart. Depending on the patient's findings, management may range from routine follow-up to liver transplantation. Here, we present a case of hypothyroidism, hepatomegaly, and cardiac failure in a two-month-old female newborn with infantile hepatic hemangioma. The patient's symptoms were managed with the use of levothyroxine, propranolol, and transcatheter arterial embolization (TAE).

## Introduction

Infantile hepatic hemangioma (IHH) is a benign liver tumor that commonly affects fetuses and newborns, with a slight female predominance. About 90% of IHH cases are identified in the first six months of age, with 30% found during the first month [1]. When IHH was initially discovered in 1919, its varied clinical symptomatology was a common cause for identification. In 1971, based on the size and vascularity of the tumor, the early cases of IHHs were divided into two types: type 1 and type 2 [2]. Only a small percentage of IHHs are type 2, which may be cancerous, but type 1 is the most common variety and is generally considered to be benign [3]. IHHs were further divided into symptomatic and asymptomatic variants in 2004 according to the size of the tumor and its hemodynamic features [4]. In 2007, a systematic categorization scheme was devised for IHH lesions based on the clinical symptoms, history of patients, and radiological and pathological features, dividing lesions into focal, multifocal, and diffuse categories [5]. Radiological investigations (ultrasonography, computed tomography (CT) scan, and magnetic resonance imaging (MRI)) are often used to diagnose IHHs. However, a biopsy is not recommended because of the significant bleeding risk. In children, small and asymptomatic lesions may resolve without any interventions. However, larger or symptomatic lesions require active treatment to prevent severe complications like cardiac failure, hepatic failure, and hypothyroidism, which can lead to mortality. Treatment options for IHHs include steroids, propranolol, and antiangiogenic drugs like interferon alpha, cyclophosphamide, and vincristine, as well as radiotherapy, selective embolization, and surgery. Transcatheter arterial embolization (TAE) is an excellent therapy for hepatic hemangiomas that reduces shunts and prevents cardiac failure [6]. In the present case, a two-month-old female child who presented with abdominal distension and jaundice was treated with propranolol and TAE but eventually succumbed to death during the postoperative period.

## Case presentation

**Figure 1:**
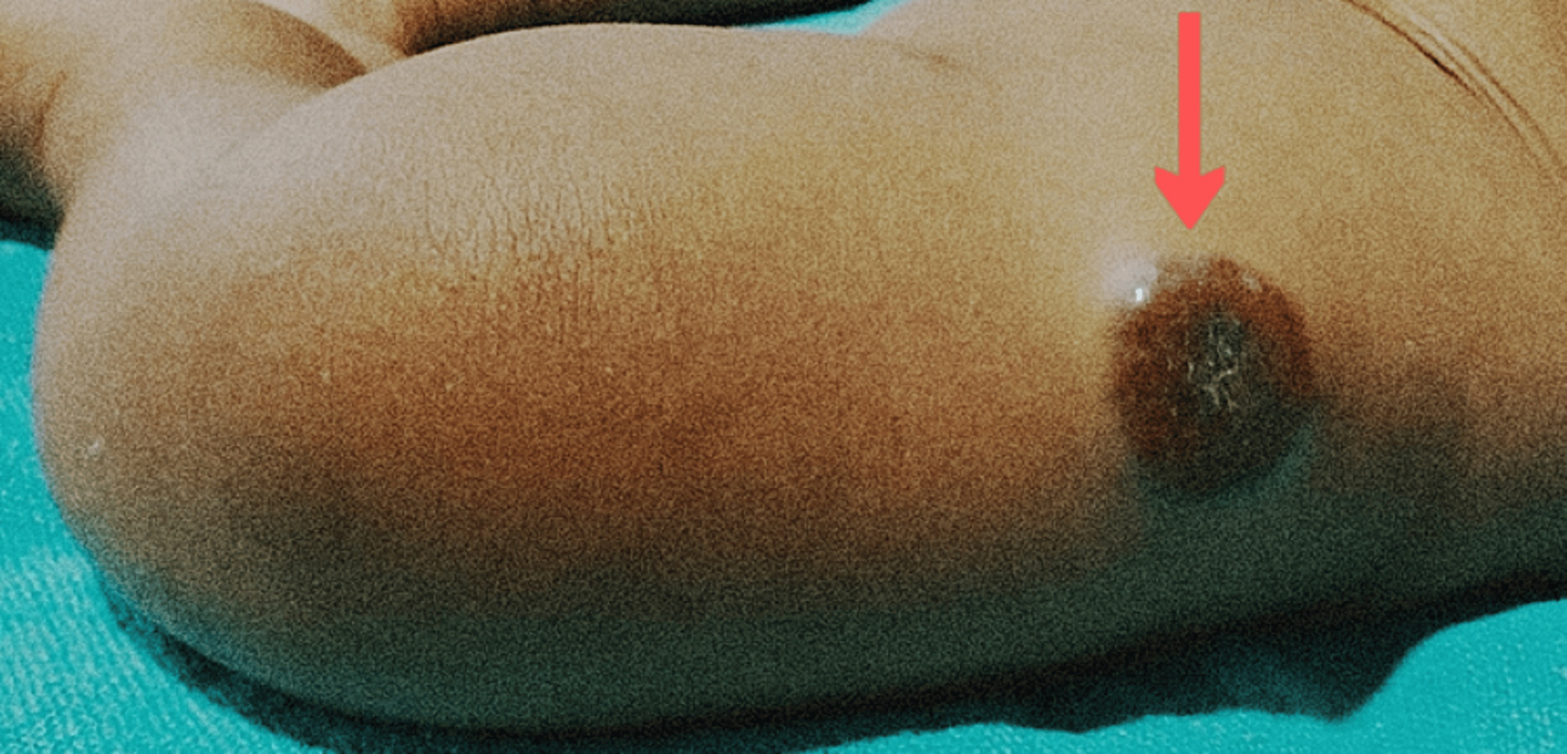
Subcutaneous hemangioma of size 3 x 3 cm on anterolateral aspect of left thigh

**Figure 2:**
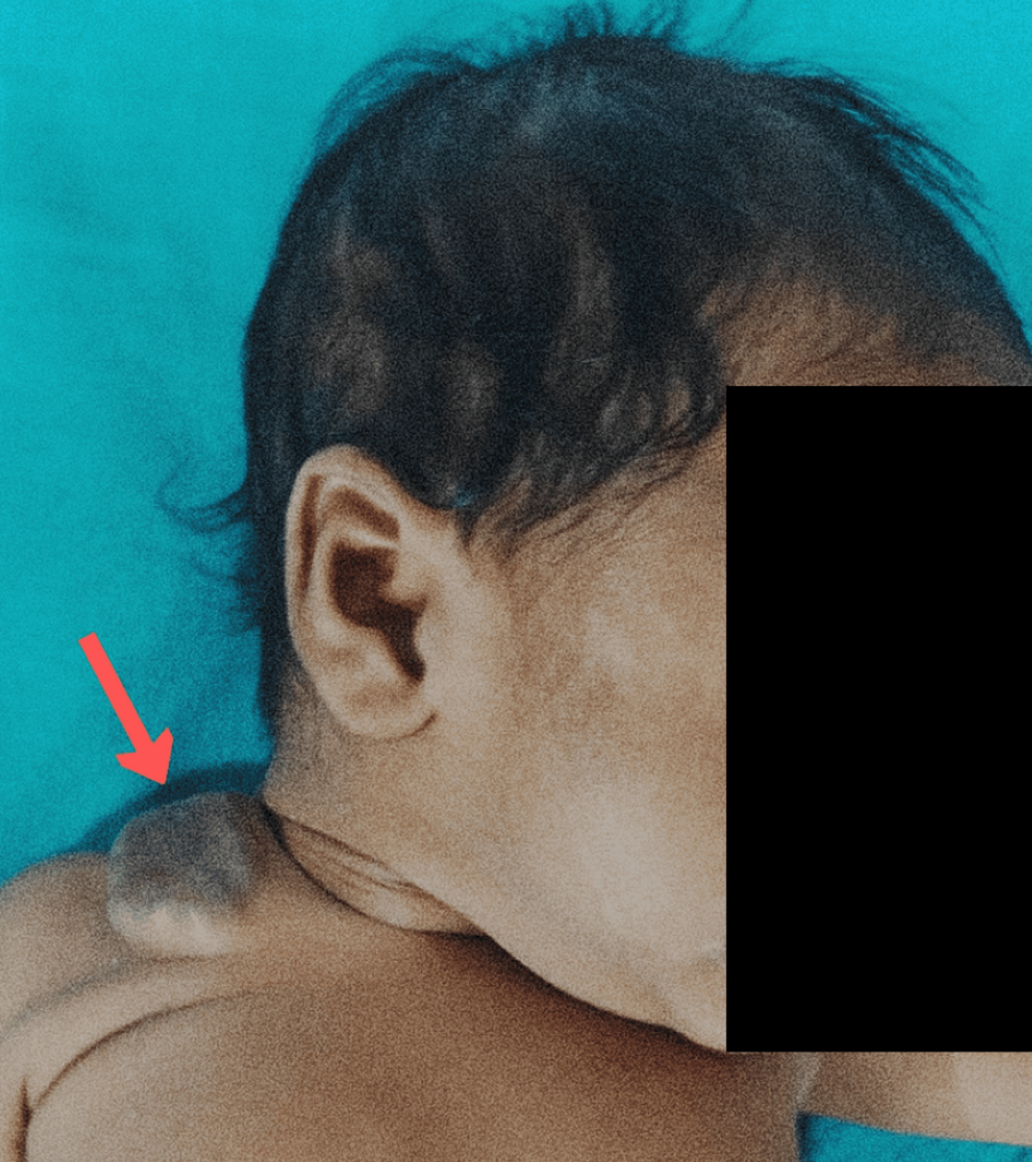
Subcutaneous hemangioma of size 3 x 3 cm in supraclavicular region

A two-month-old female child was brought to the Emergency Department with complaints of yellowish discoloration of the eye, skin, stool, and urine, along with abdominal distention and feeding difficulty for the last four days. According to the mother, the child was apparently asymptomatic five days ago when she first had discoloration of the skin, eye, urine, and stool, which started mildly and increased over time. The child had multiple episodes of melena. She was born from a non-consanguineous marriage, delivered vaginally, weighing 2,085 grams. The pregnancy proceeded normally, and there were no obstetric issues. The postpartum period was reported to be normal. There was no history of fever, cough, cold, hypertension, or jaundice. There was no history of major medical or surgical illness, nor any history of blood transfusion. The child was irritable and had mild pallor and jaundice. Subcostal retractions were also noted. The first and second heart sounds were normal, and no murmurs were detected. Upon examining the abdomen, slight distention and an enlarged liver span were observed, while the spleen was of normal size and bowel sounds were normal. No orofacial clefts or other abnormalities were found. In addition to the physical examination, no neural tube abnormalities were detected. Cutaneous hemangiomas were noted on the left lower limb (Figure 1) and right supraclavicular region (Figure 2).

**Figure 3:**
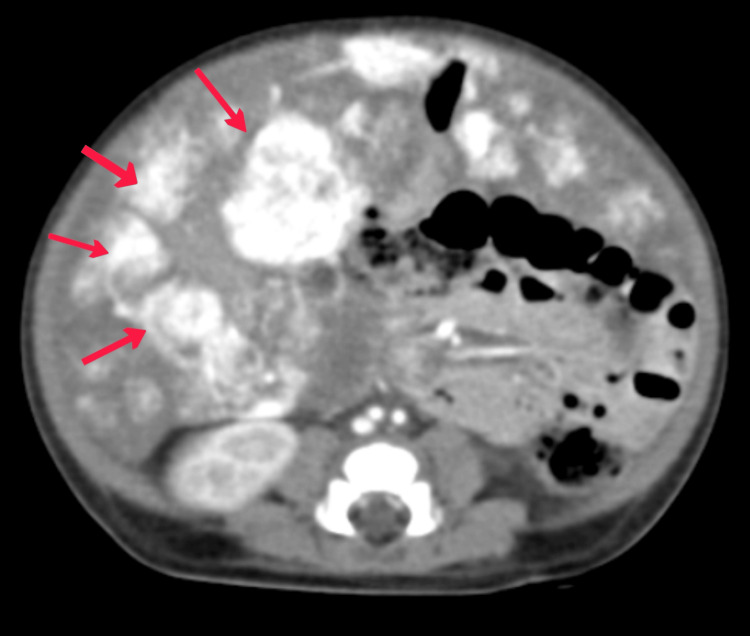
CT scan shows multiple large multilobulated mass CT: Computed tomography

She was kept on nasal prongs. An abdominal ultrasound was done to evaluate the distention. It revealed an enlarged liver with many heterogeneously hypoechoic oval-shaped lesions, the largest of which measured 3.70 x 3.80 cm in the left lobe of the liver, with cystic spaces inside it. Peripheral vascular flow was increased. A CT scan (plain and contrast-enhanced) of the upper abdomen was done to fully describe the tumor before deciding on a course of therapy. There was a 3.9 x 4 x 3.6 cm mass in the lateral section of the liver's left lobe (Figure 3). Multiple stippled calcifications were visible in it. In the portal venous phase, it showed strong nodular peripheral arterial enhancement and partial centripetal fill-in. Both the hepatic artery and the celiac trunk were normal. The aortic filling was normal above and below the celiac origin. An echocardiogram revealed a structurally normal heart without any abnormalities.

Routine laboratory investigations performed on admission are demonstrated in Table 1. They showed a free T3 level of 3.2 ng/dL, a free T4 level of 7.7 mcg/dL, and a thyroid-stimulating hormone (TSH) level of 90.6 µIU/mL, suggestive of consumptive hypothyroidism. Liver function tests were also deranged, with a serum glutamic oxaloacetic transaminase (SGOT) level of 264 U/L, a serum glutamic pyruvic transaminase (SGPT) level of 34 U/L, and an alkaline phosphatase (ALP) level of 95 U/L.

**Table 1:** Laboratory Investigations done on the day of admission TLC: Total leucocyte count; MCV: Mean corpuscular volume; MCH: Mean corpuscular hemoglobin; MCHC: Mean corpuscular hemoglobin concentration; SGOT: Serum glutamic oxaloacetic transaminase; SGPT: Serum glutamic pyruvic transaminase; ALP: Alkaline phosphatase; PT: Prothrombin time; INR: International normalized ratio; aPTT: Activated partial thromboplastin time; TSH: Thyroid stimulating hormone; FT3: Free triiodothyronine; FT4: Free thyroxine; LDH: Lactate dehydrogenase

Sr No.	Investigations	Observed value	References value
1	Hemoglobin	11.6 g/dL	14.0-23.0 g/dL
2	Hematocrit	42.9%	44.0-68.0%
3	TLC	12240 cells/cumm	9,500-28,000 cells/cumm
4	Platelet	433,000 cells/cumm	240,000-375,000 cell/cumm
5	MCV	74.8 fL	95.0-121.0 fL
6	MCH	22.8 pg	31.3-37.3 pg
7	MCHC	32.0 g/dL	29.3-37.2 g/dL
8	Total bilirubin	22.9 mg/dL	<8.5 mg/dL
9	Direct bilirubin	10.9 mg/dL	<0.60 mg/dL
10	Total protein	3.9 g/dL	4.5-7.5 g/dL
11	Albumin	2.4 g/dL	3.2-3.8 g/dL
12	Globulin	1.5 g/dL	3.2-3.6 g/dL
13	SGOT	264 U/L	46-145 U/L
14	SGPT	34 U/L	15-45 U/L
15	ALP	95 U/L	155-425 U/L
16	PT	25.8 s	10.1-15.7 s
17	INR	2.27 IU	0.54-1.61 IU
18	aPTT	56.2 s	29.5 s
19	Urea	15 mg/dL	19-43 mg/dL
20	Sodium	133 mEq/L	130-140 mEq/L
21	Creatinine	0.2 mg/dL	0.2-0.9 mg/dL
22	Potassium	5.3 mEq/L	3.5-5.0 mEq/L
23	TSH	90.6 µIU/mL	0.68-15.5 µIU/mL
24	FT3	3.2 ng/dL	0.84-2.50 ng/dL
25	FT4	7.7 pg/mL	1.70-6.5 mcg/mL
26	LDH	505 U/L	140-280 U/L

The child was started on intravenous fluids, Inj cefotaxime, and Inj vitamin K. After consulting with a dermatologist and cardiologist at our institute, the child was started on oral propranolol (1 mg/kg once a day) with close monitoring of blood pressure, pulse rate, and blood glucose levels. Within two days of starting propranolol, the treatment was well tolerated, and there were no known adverse effects. After consulting the pediatric endocrinologists, levothyroxine was started at a dosage of 9.3 μg/kg once a day. Multiple blood transfusions (fresh frozen plasma (FFP) and random donor platelets) were given preoperatively. Despite the child's clear diagnosis and worsening health, his parents agreed to TAE. The Acharya Vinoba Bhave Rural Hospital Wardha's Ethical Committee also approved the TAE. Six days after being admitted, the child underwent TAE while sedated. The patient was then moved to the operating theater (OT) table. After cleaning, painting, and draping of the perineum, 100 IU/kg of heparin was injected via the femoral artery using the Seldinger method to prevent thrombosis. The left feeding artery was embolized with gelatin sponge particles (350-560 µm). The injection was stopped when the entire amount was injected or a small branch of the portal vein around the tumor appeared. Once the embolization was complete, the microcatheter was removed, and the sheath was taken off. The femoral artery was then manually compressed for 10 to 15 minutes to confirm hemostasis. The procedure went uneventfully with minimal blood loss.

The patient was shifted to the pediatric intensive care unit (ICU) for further monitoring and postoperative care. Postoperatively, on the third day, an ultrasound of the abdomen was done, which showed an enlarged left lobe of the liver with multiple heterogeneously hyperechoic lesions. The child was transfused with FFP, as his coagulation profile was deranged (international normalized ratio (INR) 2.27, prothrombin time (PT) 25.8, activated partial thromboplastin time (aPTT) 56.2). The child appeared sick and had respiratory distress, abdominal distention, yellowish discoloration, and decreased intake of feeds. The baby was intubated but had multiple episodes of bradycardia and desaturation. The patient eventually died due to multiorgan failure on postoperative day 11.

## Discussion

IHH is the most common benign liver tumor in neonates, accounting for 12% to 20% of all cases, with the liver being the most common extracutaneous site. IHHs are asymptomatic in almost all cases. Rapidly growing masses have the potential to compress the inferior vena cava, which might result in multi-organ failure, pulmonary changes, subsequent hepatic failure, hepatomegaly, or abdominal distention. The formation of arteriovenous shunts inside these highly vascularized tumors might result in severe hypervolemia and potentially fatal congestive heart failure (CHF). Multiple lesions may also cause platelets to become trapped inside the tumors, leading to consumptive coagulopathy, thrombocytopenia, and anemia. Most hepatic hemangiomas are asymptomatic and go unnoticed, while others can cause anemia, coagulation disorders, nausea, compartment syndrome, and cardiac failure [5].

The pathophysiology of CHF in IHHs is linked to arteriovenous shunts in the hemangiomas. Diffuse and multifocal IHHs may be more likely to be recognized, and CHF may occur as a result of reduced contraction and increased cardiac output [7]. Decreased peripheral vascular resistance is the primary cause of high-output heart failure. Large arteriovenous shunts increase the amount of blood required for the vascular bed's perfusion, which raises cardiac output. In addition, the adverse effects of hypothyroidism on cardiac functioning may be associated with high-output heart failure. Increased cardiac output results from both the pulmonary and systemic blood volumes being reduced by arteriovenous shunts. Furthermore, this ultimately results in high-output CHF, which is exacerbated by pulmonary hypertension. High vascular resistance in pulmonary arteries and high pulmonary pressure during the fetal stage help maintain fetal circulation. However, following delivery, the high pulmonary pressure will progressively decrease over the course of three months, while the newborn's systemic pressure will rise as the oral foramen closes. The presence of IHHs may worsen the right heart's load and affect the transition from fetal to neonatal circulation, which, in turn, increases vascular resistance and results in pulmonary hypertension [8].

Prenatal examination effectively detects fetal abdominal masses. Imaging is necessary to diagnose these types of lesions. Ultrasound is commonly used to detect abdominal masses during pregnancy. Ultrasound reveals a hypoechoic lesion with significant blood flow. On injecting contrast agents into the vein, CT scanning detects growth in a low-density region. The enhancement begins at the perimeter and progresses to the center. After a short time, the focal region becomes as dense as the liver. While MRI takes longer than CT or ultrasound, its great spatial resolution allows for accurate lesion detection [9]. To avoid excessive sedation, we keep the child awake (for around four hours prior to the test) and administer a chloral hydrate enema. Parents prefer this alternative to anesthesia [10]. MRI scans consist of hepatic T2-weighted and axial T1-weighted images, with and without fatty saturation, as well as contrast-enhanced dynamic T1-weighted images following contrast injection. On T1-weighted scans, the lesions are hypointense in relation to the liver, while on T2-weighted scans, they are hyperintense. Hemorrhage and central necrosis can be seen in some extensive lesions. The MRI or CT scan can confirm the diagnosis. Additional investigation, such as an ultrasound-guided biopsy, laparoscopic evaluation, or technetium-99m-labeled red blood cell scans, should be considered if the child's symptoms and imaging results are not typical. These procedures are all invasive, and some hepatic hemangiomas might result in potentially fatal side effects, including hypothyroidism and high-output heart failure [9].

Hepatic hemangioma treatment commonly includes embolization, pharmacological therapy, surgical excision, and monitoring. On the other hand, there are no established treatment methods for IHHs. For a patient who presents with no symptoms, treatment is not required for many minor hepatic hemangiomas. Careful observation and monitoring are advised when there is no hepatomegaly, no sign of impending heart failure, or scan results that show fast-flow macrovascular shunting. The majority of IHH cases have spontaneous regression and are asymptomatic, so those who develop heart failure have a significant risk of death [11]. The administration of medical therapy to patients should follow the same guidelines as for problematic cutaneous hemangiomas in the absence of any other condition. It has been documented that propranolol is an effective treatment for IHHs [12].

Since 2008, propranolol has been the preferred initial treatment for rapidly growing hemangiomas, attributed to its high efficacy and minimal side effects [11]. The FDA recommends 0.60 mg/kg twice a day as the starting dose of propranolol, and over the course of two weeks, this amount should be gradually increased to 1.7 mg/kg twice daily. According to a European expert consensus committee, propranolol should be started at 1.0 mg/kg per day, with a target dosage of 2.0 to 3.0 mg/kg per day, split into two or three doses. The patient's condition should be considered while adjusting the dose, particularly if the patient has adverse reactions or coarctation of the aorta, hemangioma, arterial anomalies, or abnormalities of the eyes. The course of treatment should last for at least 6 months and up to 12 months. Nearly 10% to 25% of individuals may experience rebound growth during the tapering phase or withdrawal period; this can happen even six months after the discontinuation of the medication [13,14]. Propranolol side effects are uncommon in both cutaneous and hepatic hemangiomas. Typical side effects include bradycardia, acrocyanosis, hypotension, and disturbed sleep. Most negative effects are temporary and occur one to three hours following oral medication. Reports of bronchospasm, bradycardia, hypoglycemia, symptomatic hypotension, and even hypoglycemic seizures are examples of serious adverse effects, though they are uncommon. Propranolol should not be used during periods of severe respiratory illness or bronchial spasms, and prolonged fasting is not recommended [15]. Digoxin, angiotensin-converting enzyme inhibitors, and diuretics may also be given to individuals who are in high-output heart failure. Furthermore, patients with hemocytopenia may require blood component transfusions. High levels of D3 iodothyronine deiodinase activity are seen in IHH tissue, and this enzyme can convert T4 and T3 into inactive metabolites. Consumptive hypothyroidism may develop if the IHH consumes most of the liver tissue [9].

The required dosage varies per person; in this case, a dose of 9.5 μg/kg levothyroxine once a day (or 37.45 μg/day) produced a quick and long-lasting effect. Significantly higher doses have been documented in the literature, such as a case where a female child with IHH and consumptive hypothyroidism was treated with 75 μg/day of levothyroxine [16]. Second-line treatments for cases of resistance include vincristine, corticosteroids, and interferon [11]. Infants in critical condition who need vasopressor treatment or mechanical breathing may be considered for embolization. TAE is an effective therapy for hemangioma shunt reduction. When high-output CHF and arteriovenous malformations aggravate IHHs, embolization may be considered. Prior to hepatic lesion embolization, careful examination using superior mesenteric arteriography to show the venous phase of the portal vein and aortography to concentrate on the systemic vascular supply is recommended. Furthermore, it is preferable to operate in stages to reduce the risk of liver necrosis and mortality [17]. Hepatic artery ligation (HAL) or hepatic artery embolization (HAE) is a successful alternative strategy to reduce shunts and prevent heart failure. Due to the unsustainable nature of complete hepatectomy, individuals with IHHs may potentially opt for orthotopic liver transplantation [17].

## Conclusions

For the diagnosis of IHHs, appropriate imaging techniques and prenatal diagnostics are essential. Adequate treatment time is required for the disease to be cured. Patients with IHHs who are asymptomatic should be closely monitored. Propranolol is thought to work well for uncomplicated hemangiomas as well. Treatment for IHHs with potentially fatal outcomes includes propranolol and/or TAE. It is crucial to emphasize that early, aggressive treatment is essential in symptomatic IHH patients, even though most cases of IHHs are asymptomatic and spontaneously resolve. In IHHs, embolization should be carefully considered. Embolization or ablation are minimally invasive interventional radiology procedures that are appropriate as initial treatments when therapy is necessary due to life-altering symptoms since they have a lower risk of bleeding than surgery. However, hepatic surgical resection or enucleation remains the gold standard for treating these refractory patients if embolization or ablative therapy fails. In the upcoming years, more multicenter randomized trials must be conducted. Insufficient information may result in care that is both inadequate and inaccurate. Further details on IHHs and the best ways to treat them are required.
